# Reticulated Platelets and an Optimal Model for Differentiating Thrombocytopenia Etiologies

**DOI:** 10.1002/jha2.70308

**Published:** 2026-05-15

**Authors:** Tung Thanh Tran, Quyen Ngoc Truong, Hong Phuoc Mac, Thang Thanh Phan, Tai Chau Quach, Son Truong Nguyen

**Affiliations:** ^1^ Department of Hematology Cho Ray Hospital Ho Chi Minh City Vietnam; ^2^ Department of Molecular Biomedicine Gia An 115 Hospital Ho Chi Minh City Vietnam

**Keywords:** optimal model, reticulated platelets, thrombocytopenia

## Abstract

**Objectives:**

This study aimed to investigate the clinical value of reticulated platelets (RtcPLT) percentage derived from an automated hematology analyzer in differentiating thrombocytopenia due to peripheral etiology from decreased bone marrow production (BME). In addition, a secondary objective was to establish specific reference intervals for RtcPLT and to propose an optimal diagnostic model.

**Methods:**

We conducted a descriptive cross‐sectional study of 375 subjects, including 84 healthy controls and 291 thrombocytopenic patients. The healthy individuals were utilized to establish the RtcPLT reference range. The patient cohort was classified into PE and BME groups based on the results of bone marrow aspiration and biopsy (BMAB). RtcPLT was measured using the optical fluorescence method on the ADVIA 2120i platform. Diagnostic accuracy was evaluated using receiver operating characteristic (ROC) analysis and Bayesian model averaging (BMA) to propose the optimal model.

**Results:**

The median RtcPLT in the BME group (2.28%) was significantly lower than that of the PE group (8.27%, *p* < 0.001). The single RtcPLT index (cutoff 4.64%) achieved an area under the curve (AUC) of 0.895 (95% confidence interval [95% CI]: 0.858–0.932), showing high sensitivity (90.7%, 95% CI: 81.7%–96.2%), specitificity (82.9%, 95% CI: 77.2%–87.6%) and negative predictive value (NPV) (NPV: 96.2%, 95% CI: 92.4%–98.5%). The optimal model, combining RtcPLT with red blood cells (RBC), neutrophil%, basophil%, retic%, and large unstained cells (LUC%), achieved superior accuracy: AUC value of 0.980 (95% CI: 0.967–0.993), sensitivity of 92.0% (95% CI: 83.4%–97.0%), and specificity of 94.4% (95% CI: 90.5%–97.1%).

**Conclusion:**

The RtcPLT index measured in this study is a robust, rapid, and automated biomarker for assessing thrombopoietic activity. Furthermore, the optimal model we suggested provides near‐perfect diagnostic accuracy, serving as an expeditious and cost‐effective screening tool to reliably differentiate peripheral and bone marrow etiologies of thrombocytopenia.

**Trial Registration:**

The authors have confirmed clinical trial registration is not needed for this submission

**Abbreviations**
95% CI: 95% confidence interval
AUC: area under the curve
BMA: Bayesian model averaging
BMAB: bone marrow aspiration and biopsy
BME: bone marrow etiology
CBC: complete blood count
FAB: French‐American‐British
HC: healthy controls
HCT: hemetorit
HGB: hemoglobin
IPF: immature platelet fraction
ITP: immune thrombocytopenia/Immune thrombocytopenic purpura
LUC: large unstained cells
MCH: mean corpuscular hemoglobin
MCV: mean corpuscular volume
MPV: mean platelet volume
NPV: negative predictive value
PE: peripheral etiology
PLT: platelets
PPV: positive predictive value
RBC: red blood cells
Retic: reticulocyte
ROC: receiver operating characteristic
RtcPLT: reticulated platelets
ULN: upper limit of normal
WBC: white blood cells
WHO: World Health Organization

## 1 Introduction

Thrombocytopenia, the second most common hematological disorder, requires prompt etiological diagnosis as it predisposes patients to life‐threatening hemorrhage. This condition is broadly categorized into decreased production in the bone marrow (bone marrow etiology [BME]) or increased peripheral destruction/consumption (peripheral etiology [PE]) [1, 2]. Distinguishing between these mechanisms is crucial for therapeutic decisions, yet the current gold standard, bone marrow aspiration and biopsy (BMAB), is invasive, time‐consuming, and costly [1, 3]. Reticulated platelets (RtcPLT), or immature platelet fraction (IPF), are newly formed platelets (PLT) reflecting megakaryocyte activity, acting as a potential noninvasive indicator, typically showing markedly elevated values in PE and low values in BME. Numerous studies have consistently affirmed RtcPLT/IPF as a reliable parameter for the differential diagnosis of thrombocytopenia, demonstrating elevated levels in patients with peripheral etiology (PE) compared to those with decreased production (BME) [2, 3, 4]. However, despite this consensus, diagnostic thresholds for RtcPLT/IPF vary significantly across instrument platforms due to differences in staining methodologies (e.g., Sysmex vs. other systems). Given the comparatively limited data validating RtcPLT indices on this specific analyzer platform [5], and recognizing that a single biomarker may not capture the multilineage dysfunction often present in BME [6, 7]. This study was designed specifically to validate the RtcPLT index and to suggest an optimal model by combining the RtcPLT value with other routine hematological parameters, aiming to provide a superior, rapid, and cost‐effective screening tool for reliable differential diagnosis of thrombocytopenia etiologies.

## 2 Materials and Methods

### 2.1 Study Design and Subjects Selection

This research employed a descriptive cross‐sectional study design, utilizing data collected from July 2023 to May 2024 at Cho Ray Hospital. The cohort included 375 subjects, comprising 84 healthy controls (HC) and 291 eligible patients presenting with thrombocytopenia (PLT < 150 G/L). All HC were confirmed to be free from any medical conditions and were not taking any medications. Patients with evidence of active infection, hepatic disease, splenomegaly, or thrombocytopenia attributable to drug use were excluded, as these conditions may independently alter platelet turnover and confound RtcPLT measurements. Patients were excluded if they had received therapeutic intervention or platelet transfusion within the preceding 48 h to avoid confounding RtcPLT results. The final 291 patients were definitively classified into BME (*n* = 216) and PE (*n* = 75) groups based on bone marrow smear results and comprehensive medical records. The HC group was utilized to establish the normal reference range for RtcPLT value.

The study was reviewed and approved by the Ethics Committee Board at Cho Ray Hospital on September 9, 2023, under number 631/GCN‐HDDD. This study does not interfere with routine treatment or clinical examination decisions. The researchers commit to maintaining the confidentiality of all information collected from the research subjects.

### 2.2 Bone Marrow Assessment and Reticulated Platelets Analysis

BMAB was performed in all 291 thrombocytopenic patients as part of the routine diagnostic evaluation and served as the reference standard for patient classification into BME and PE groups. BMAB was performed to examine cell morphology and evaluate the rate of hematopoiesis. Bone marrow smears were stained with Wright–Giemsa solution and examined by two independent cytopathologists. Final diagnosis relied on established French‐American‐British (FAB) 1976 [8] and World Health Organization (WHO) 2022 classifications [9], assessing morphological features, hematopoietic cell lines, and megakaryocyte counts [10].

**FIGURE 1:**
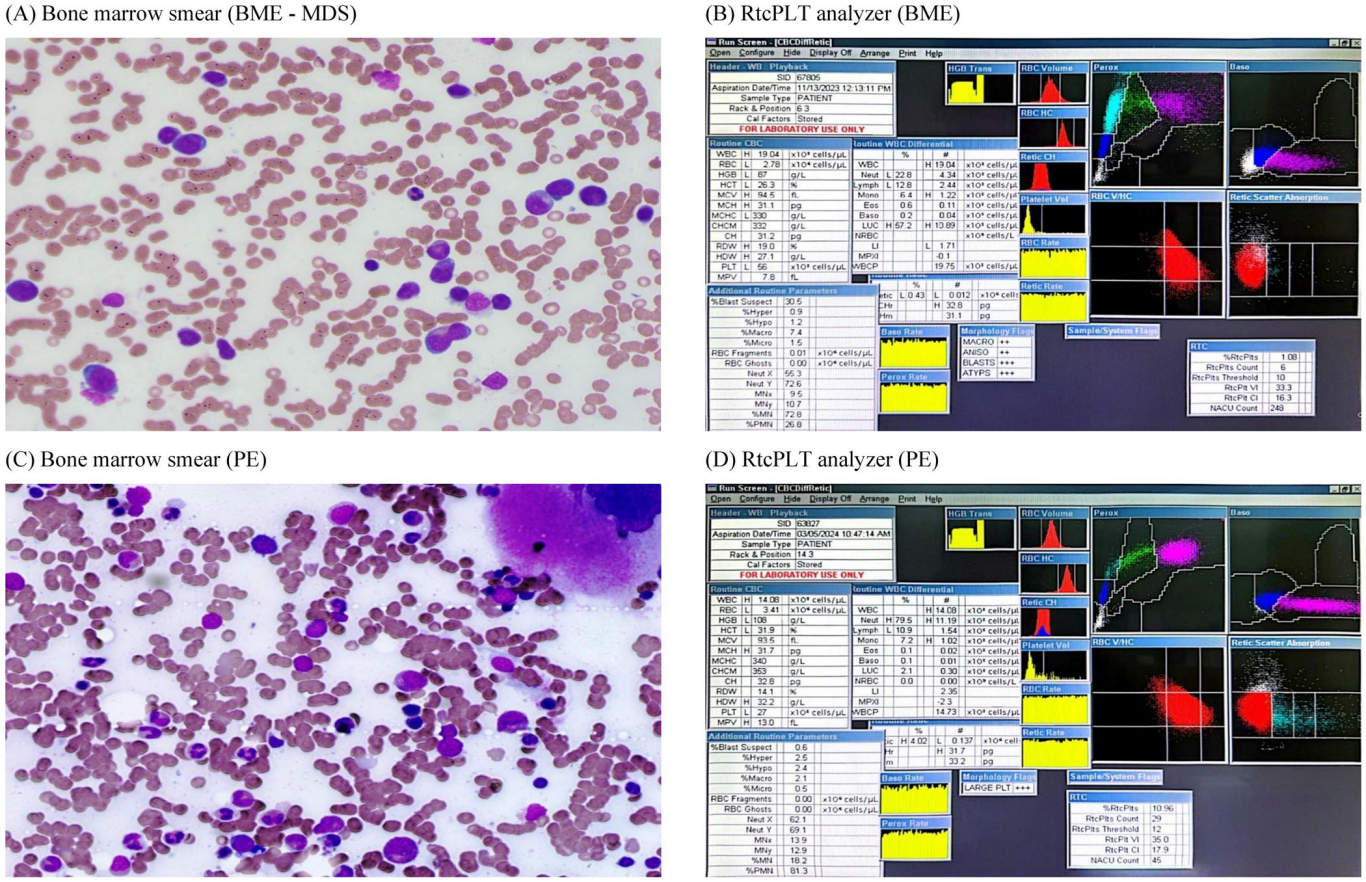
Representative bone marrow morphology and reticulated platelet analysis in thrombocytopenia. (A) Bone marrow smear of a case with myelodysplastic syndrome. (B) Reticulated platelets of a case with myelodysplastic syndrome. (C) Bone marrow smear of a case with peripheral thrombocytopenia. (D) Reticulated platelets of a case of peripheral thrombocytopenia.

The RtcPLT percentage was measured automatically using the ADVIA 2120i hematology analyzer (Siemens Healthineers, Figure 1). This system employs an optical fluorescence method in the reticulocyte channel, utilizing polymethine dye to stain the RNA content of newly released PLT. The RtcPLT value, along with the other parameters used for the optimal model, was derived from the complete blood count (CBC) test generated by the same analyzer. The RtcPLT reference interval was established from the 84 HC using the robust statistical method, in accordance with CLSI guideline EP28‐A3c, which endorses this approach for reference sample groups of 20–120 subjects.

### 2.3 Statistical Analysis

Statistical analysis was conducted using R software (R Foundation, Austria), with a two‐sided significance level set at *p* < 0.05. Given the non‐normal distribution of continuous hematological data, results were presented as median and the 95% confidence interval (95% CI), and the non‐parametric Kruskal–Wallis rank test was used for group comparisons. The receiver operating characteristic (ROC) curve analysis was performed to evaluate the univariate diagnostic power of RtcPLT value and other parameters, with the optimal cutoff determined by maximizing the Youden index. Moreover, the sensitivity, specificity, positive predictive value (PPV), and negative predictive value (NPV) were calculated. Finally, Bayesian model averaging (BMA) analysis was performed to construct the optimal model, and its performance was evaluated by its area under the curve (AUC), sensitivity, and specificity. In addition, the model's accuracy was assessed using calibration test.

## 3 Results

### 3.1 Characteristics of Study Subjects

The study analyzed data from a total of 375 subjects, including 84 HC and 291 patients with thrombocytopenia, comprising 216 BME cases and 75 PE cases (Table 1). The demographic analysis revealed no significant difference in the male‐to‐female ratio between the BME and PE groups (*p* = 0.697). However, a statistically significant difference was observed in age, with the median age in the PE group (49 years, 95% CI: 40–54) being substantially lower than that in the BME group (60 years, 95% CI: 57–62, *p* = 0.001).

**TABLE 1:** Characteristics of the included study subjects.

Variable	Healthy control (*n *= 84)	Bone marrow cause (*n* = 216)	Peripheral cause (*n* = 75)	*p*‐value^a^
Median age, year (95% CI)	36 (32–37)	60 (57–62)	49 (40–54)	0.001
Gender: female/male	58/26	103/113	39/36	0.697
RBC, 10^12^/L (95% CI)	4.67 (4.57–4.77)	2.62 (2.55–2.75)	3.60 (3.25–4.08)	< 0.001
HGB, g/L (95% CI)	134 (132–137)	82 (79–83)	105 (95–117)	< 0.001
HCT, % (95% CI)	41.0 (40.1–42.0)	24.8 (24.2–25.5)	32.3 (28.0–35.4)	< 0.001
MCV, fL (95% CI)	88.9 (87.9–89.6)	92.6 (91.1–94.5)	87.9 (86.4–91.1)	0.003
MCH, pg	29.0 (28.8–29.5)	30.4 (29.7–30.9)	29.1 (28.5–29.8)	0.006
Retic, % (95% CI)	1.83 (1.72–1.99)	1.23 (1.09–1.47)	2.53 (1.94–3.15)	< 0.001
WBC, 10^9^/L (95% CI)	6.72 (6.30–7.52)	7.15 (5.35–8.52)	8.78 (7.53–10.83)	0.463
Neutrophil, % (95% CI)	55.3 (53.4–57.5)	28.6 (25.3–34.2)	66.3 (63.2–71.7)	< 0.001
Lymphocyte, % (95% CI)	34.6 (32.4–35.5)	32.6 (28.3–39.0)	20.9 (18.2–23.4)	< 0.001
Monocyte, % (95% CI)	6.2 (5.8–6.8)	5.7 (4.5–6.8)	4.9 (4.4–5.6)	0.345
Eosinophil, % (95% CI)	2.3 (1.8–2.6)	0.9 (0.7–1.1)	1.3 (0.7–1.9)	0.232
Basophil, % (95% CI)	0.5 (0.4–0.5)	0.5 (0.5–0.6)	0.2 (0.2–0.3)	< 0.001
LUC, % (95% CI)	0.0 (0.0–0.1)	7.9 (6.1–10.5)	2.4 (1.8–2.8)	< 0.001
PLT, 10^9^/L (95% CI)	283 (274–298)	49 (41–55)	31 (26–35)	< 0.001
MPV, fL (95% CI)	8.2 (8.0–8.6)	8.4 (8.3–8.6)	10.0 (9.3–10.6)	< 0.001
RtcPLT, % (95% CI)	1.57 (1.37–1.81)	2.28 (1.95–2.59)	8.27 (7.01–8.82)	< 0.001

*Note*: Data were presented as median (95% CI).

Abbreviations: 95% CI, 95% confidence interval; HCT, hematocrit; HGB, hemoglobin; LUC, large unstained cells; MCH, mean corpuscular hemoglobin; MCV, mean corpuscular volume; MPV, mean platelet volume; PLT, platelets; RBC, red blood cells; RtcPLT, reticulated platelets; WBC, white blood cells.

^a^ Difference between the bone marrow and peripheral cause groups.

**FIGURE 2:**
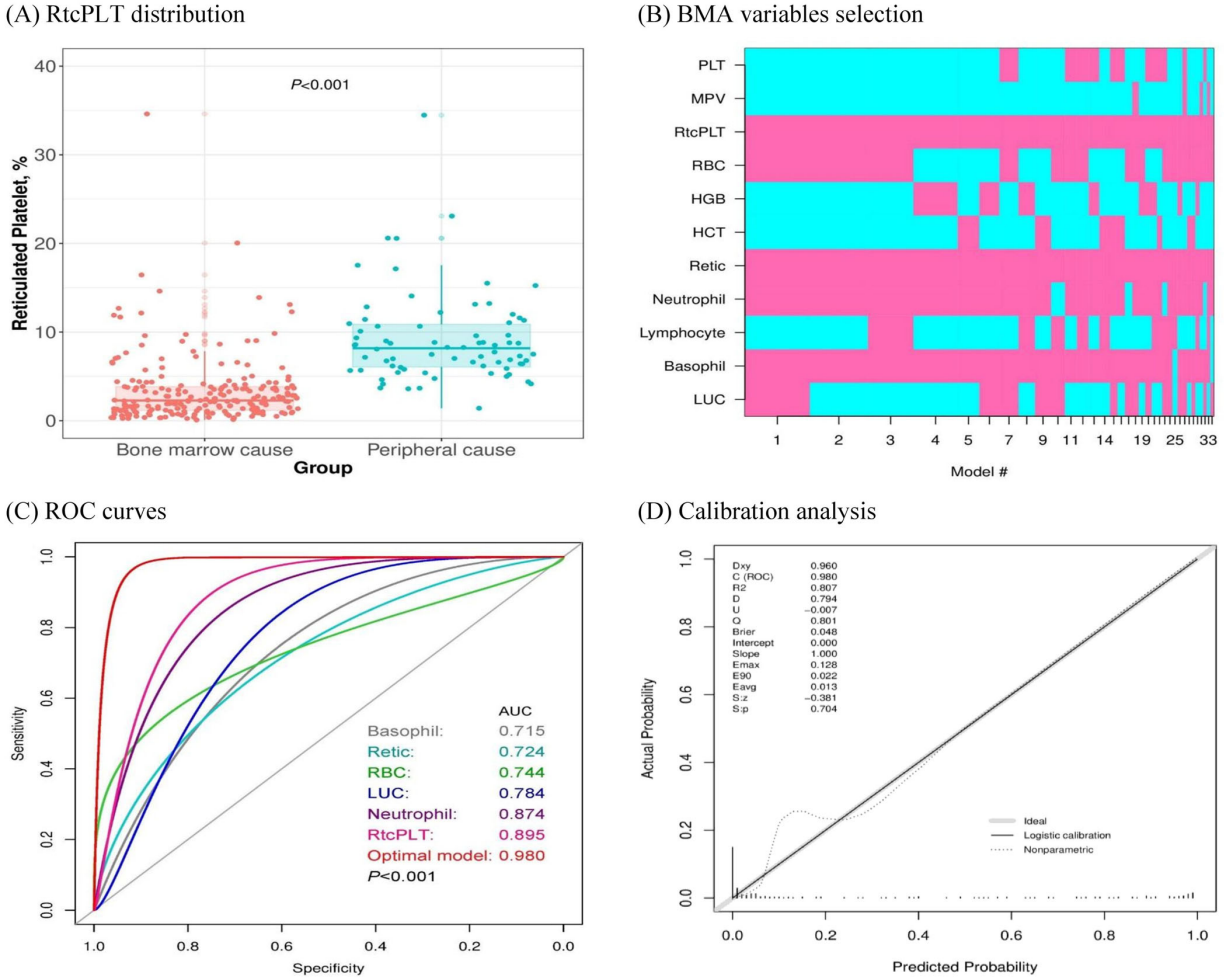
Diagnostic performance of reticulated platelets and the optimal model. (A) Distribution of reticulated platelets between groups. (B) Bayesian model averaging (BMA) statistics used to select variables for the optimal model. (C) Comparison of model performance versus individual biomarkers. (D) Calibration analysis of the optimal model.

The median RtcPLT reference interval established from the HC cohort was 1.57% (95% CI: 1.37%–1.81%). A highly significant difference was noted between the patient groups: the BME group exhibited a median RtcPLT of 2.28% (95% CI: 1.95%–2.59%), indicating suppressed thrombopoiesis, which was markedly lower than the PE group at 8.27% (95% CI: 7.01%–8.82%, *p* < 0.001, Figure 2A).

Comparative analysis of other key hematological indices reflecting multilineage involvement showed significant differences between the two patient groups (data shown in Table 1). In terms of the erythroid lineage, the BME group displayed more severe anemia, with median hemoglobin (HGB) of 82 g/L (95% CI: 79–83 g/L) compared to 105 g/L (95% CI: 95–117 g/L) in the PE group. Similarly, RBC count and hematocrit (HCT) were significantly lower in BME cases. Furthermore, the erythropoietic response, represented by reticulocyte percentage (retic%), was also significantly lower in the BME group (1.23%, 95% CI: 1.09%–1.47%) compared to the PE group (2.53%, 95% CI: 1.94%–3.15%) (*p* < 0.001). Regarding myeloid and platelet indices, significant differences were recorded in neutrophil percentage (neutrophil%) (BME: 28.6% vs. PE: 66.3%, *p* < 0.001) and large unstained cells percentage (LUC%) (BME: 7.9% vs. PE: 2.4%, *p* < 0.001). In addition, mean platelet volume (MPV) was significantly higher in the PE group (10.0 fL, 95% CI: 9.3–10.6 fL) compared to the BME group (8.4 fL, 95% CI: 8.3–8.6 fL, *p* < 0.001).

### 3.2 Performance of RtcPLT and Optimal Model in Differentiating Causes of Thrombocytopenia

ROC analysis confirmed that the RtcPLT is a highly effective standalone biomarker for discriminating between BME and PE (Figure 2C). Using an optimal cutoff point of 4.64% (determined by maximizing the Youden index), the RtcPLT index achieved a robust AUC of 0.895 (95% CI: 0.858–0.932), demonstrating high sensitivity (90.7%, 95% CI: 81.7%–96.2%), specificity (82.9%, 95% CI: 77.2%–87.6%), and notably high NPV (NPV: 96.2%, 95% CI: 92.4%–98.5%). Other hematological parameters showing strong discriminatory power included neutrophil% (AUC 0.874), RBC (AUC 0.744), and retic% (AUC 0.724), indicating the potential synergistic value of a combined index (Table 2). To maximize diagnostic certainty and account for multilineage involvement, a composite index was developed. By using BMA analysis, we developed an optimal model combining RtcPLT value with five supplementary routine CBC parameters: RBC, neutrophil%, basophil%, retic%, and LUC% (Figure 2B, Table 2). This optimal model demonstrated significantly superior performance compared to any single marker, yielding a near‐perfect AUC of 0.980 (95% CI: 0.967–0.993). The model achieved high diagnostic metrics, including a sensitivity of 92.0% (95% CI: 83.4%–97.0%) and specificity of 94.4% (95% CI: 90.5%–97.1%), confirming its robust utility in reliably differentiating the causes of thrombocytopenia (Table 2). Furthermore, calibration analysis indicates that the optimal model, demonstrated by a very low Brier index (0.048), can be effectively utilized in clinical practice (Figure 2D).

**TABLE 2:** Performance of individual markers in differentiating causes of thrombocytopenia.

Variable	Cutoff	AUC (95% CI)	Sensitivity, % (95% CI)	Specificity, % (95% CI)	PPV, % (95% CI)	NPV, % (95% CI)
RBC, 10^12^/L	≥ 3.32	0.744 (0.670–0.819)	61.3 (49.4–72.4)	82.9 (77.2–87.6)	55.4 (44.1–66.3)	86.1 (80.6–90.5)
HGB, g/L	≥ 95	0.720 (0.641–0.799)	62.7 (50.7–73.6)	81.9 (76.2–86.8)	54.7 (43.5–65.4)	86.3 (80.9–90.7)
HCT, %	≥ 29.4	0.728 (0.650–0.806)	60.0 (48.0–71.1)	83.3 (77.7–88.0)	55.6 (44.1–66.6)	85.7 (80.2–90.1)
Retic, %	≥ 1.42	0.724 (0.659–0.789)	84.0 (73.7–91.4)	55.6 (48.7–62.3)	39.6 (32.0–47.7)	90.9 (84.7–95.2)
Neutrophil, %	≥ 46.2	0.874 (0.831–0.916)	92.0 (83.4–97.0)	74.5 (68.2–80.2)	55.6 (46.5–64.6)	96.4 (92.3–98.7)
Lymphocyte, %	≤ 31.4	0.673 (0.612–0.734)	90.7 (81.7–96.2)	52.3 (45.4–59.1)	39.8 (32.4–47.5)	94.2 (88.4–97.6)
Basophil, %	≤ 0.3	0.715 (0.653–0.777)	68.0 (56.2–78.3)	65.7 (59.0–72.0)	40.8 (32.1–49.9)	85.5 (79.3–90.5)
LUC, %	≤ 3.3	0.784 (0.730–0.839)	72.0 (60.4–81.8)	76.4 (70.2–81.9)	51.4 (41.5–61.3)	88.7 (83.3–92.9)
PLT, 10^9^/L	≤ 12	0.664 (0.591–0.738)	28.0 (18.2–39.6)	98.1 (95.3–99.5)	84.0 (63.9–95.5)	79.7 (74.4–84.4)
MPV, fL	≥ 9.3	0.699 (0.627–0.771)	65.3 (53.5–76.0)	73.1 (66.7–78.9)	45.8 (36.1–55.7)	85.9 (80.0–90.6)
RtcPLT, %	≥ 4.64	0.895 (0.858–0.932)	90.7 (81.7–96.2)	82.9 (77.2–87.6)	64.8 (54.8–73.8)	96.2 (92.4–98.5)
Optimal model^a^	≥ −0.39	0.980 (0.967–0.993)	92.0 (83.4–97.0)	94.4 (90.5–97.1)	85.2 (75.6–92.1)	97.1 (93.9–98.9)

Abbreviations: 95% CI, 95% confidence interval; AUC, area under curve; HCT, hematocrit; HGB, hemoglobin; LUC, large unstained cells; MPV, mean platelet volume; NPV, negative predictive value; PLT, platelets; PPV, positive predictive value; RBC, red blood cells; RtcPLT, reticulated platelets.

^a^ *Y* = −10.3 + (0.3866 × RtcPLT) + (1.683 × RBC) + (0.2941 × retic%) + (0.0444 × neutrophil%) − (2.23 × basophil%) − (0.0974 × LUC%); only biomarkers with AUC ≥ 0.65 are shown.

## 4 Discussion

Thrombocytopenia is a pervasive hematological challenge where accurate differentiation between BME and PE is critical for timely therapeutic intervention [1, 2]. The pathophysiology of thrombocytopenia is categorized based on the underlying mechanism, whether it is a primary failure of megakaryopoiesis in the bone marrow (BME, seen in leukemias, myelodysplastic syndrome, or aplastic anemia) or accelerated clearance/consumption in the periphery (PE, characteristic of immune thrombocytopenia or immune thrombocytopenic purpura [ITP]) [11]. The distinction between these two broad categories is paramount because treatment strategies diverge fundamentally. For PE, therapies often involve immunosuppression (e.g., in ITP), while BME requires specific oncological or supportive intervention, often guided by the detailed classifications provided in the WHO 2022 classification of hematolymphoid tumors [9]. The use of rapid, noninvasive tools to correctly stratify patients is crucial for adhering to the latest diagnostic and management guidelines, thereby improving patient outcomes and reducing diagnostic delay.

Both IPF and RtcPLT are increasingly recognized as valuable laboratory indices for the differential diagnosis of thrombocytopenia, offering a noninvasive alternative that can potentially minimize the need for invasive bone marrow procedures [12, 13]. While the clinical utility of IPF has been extensively investigated [14, 15], research focusing specifically on RtcPLT remains comparatively limited [16, 17]. Previous reports have demonstrated that RtcPLT quantification via flow cytometry possesses good discriminatory capability [18, 19]; however, this method is constrained by relatively high costs, technical complexity, and longer turnaround times compared to routine CBC. Furthermore, there is a scarcity of studies evaluating the clinical application of RtcPLT parameters derived directly from automated hematology analyzers, underscoring the necessity for further validation on platforms such as the ADVIA system [20, 21].

We conducted a cross‐sectional study at Cho Ray Hospital and found that RtcPLT values were higher in patients with PE compared to those with BME thrombocytopenia and HC. The RtcPLT index alone demonstrated strong discriminative performance in differentiating the etiologies of thrombocytopenia. Furthermore, a multivariate model combining RtcPLT with routine hematological parameters (RBC, neutrophil%, basophil%, retic%, and LUC%) achieved significantly greater diagnostic efficiency in distinguishing BME from PE. A particularly important finding from our ROC analysis using the Youden index was the identification of an optimal RtcPLT cutoff of 4.64%, yielding an AUC of 0.895, which clearly outperformed the AUC value of conventional mean + 2SD (upper limit of normal [ULN]) approach recommended on previous guidelines [22, 23]. When the ULN‐based threshold of 3.21% was applied to our Vietnamese cohort, the AUC decreased to 0.810, indicating a noticeable loss of diagnostic performance and demonstrating that this statistically derived cutoff did not maximize the discriminative potential of RtcPLT in our setting. These results raise a critical question as to whether a single ULN‐based threshold can be universally applied across different populations, ethnic groups, and disease spectra worldwide. While further data are needed to provide a definitive answer, our findings strongly suggest that each center should establish and validate its own local RtcPLT cutoff values, tailored to its patient population and analytical platform, in order to optimize diagnostic accuracy and clinical decision‐making in routine practice.

This study is limited by its cross‐sectional, single‐center design, meaning the quantitative thresholds established (like the RtcPLT cutoff of 4.64% and the optimal model coefficients) may require cautious generalization to other patient populations or countries with different disease prevalence rates. Future multicenter, prospective validation studies are strongly recommended to externally test and confirm the robustness and transportability of the optimal multivariate index across varied clinical environments.

## 5 Conclusions

In summary, the RtcPLT index was significantly higher in the PE group compared to both the BME thrombocytopenia group and HC. In addition, RtcPLT demonstrated high accuracy, sensitivity, and specificity in differentiating PE from BME. The combination of RtcPLT with conventional hematological parameters further improved its diagnostic performance in clinical settings.

## Author Contributions

Tung Thanh Tran and Son Truong Nguyen are senior authors who contributed to the study design and resources. Tai Chau Quach selected patients for the study. Quyen Ngoc Truong performed CBC testing and collected data. Thang Thanh Phan and Hong Phuoc Mac analyzed data and wrote the manuscript. All authors reviewed and approved the final version of the manuscript.

## Funding

The authors have nothing to report.

## Ethics Statement

This study was reviewed and approved by the Ethics Committee Board at Cho Ray Hospital on September 9, 2023, under number 631/GCN‐HDDD.

## Consent

Following the routine medical examination procedure, patients were counseled and asked to provide written informed consent before performing the bone marrow aspiration. Peripheral blood samples used in the RtcPLT analysis were obtained after routine CBC testing. Investigators are responsible for the confidentiality of all participants' personal information.

## Conflicts of Interest

The authors declare no conflicts of interest.

## Data Availability

The data that support the findings of this study are available from the corresponding author upon reasonable request.
